# Singing modulates parvalbumin interneurons throughout songbird forebrain vocal control circuitry

**DOI:** 10.1371/journal.pone.0172944

**Published:** 2017-02-24

**Authors:** Yildiz Zengin-Toktas, Sarah Cushing Woolley

**Affiliations:** Department of Biology, McGill University, Montreal, Québec, Canada; Texas Christian University, UNITED STATES

## Abstract

Across species, the performance of vocal signals can be modulated by the social environment. Zebra finches, for example, adjust their song performance when singing to females (‘female-directed’ or FD song) compared to when singing in isolation (‘undirected’ or UD song). These changes are salient, as females prefer the FD song over the UD song. Despite the importance of these performance changes, the neural mechanisms underlying this social modulation remain poorly understood. Previous work in finches has established that expression of the immediate early gene EGR1 is increased during singing and modulated by social context within the vocal control circuitry. Here, we examined whether particular neural subpopulations within those vocal control regions exhibit similar modulations of EGR1 expression. We compared EGR1 expression in neurons expressing parvalbumin (PV), a calcium buffer that modulates network plasticity and homeostasis, among males that performed FD song, males that produced UD song, or males that did not sing. We found that, overall, singing but not social context significantly affected EGR1 expression in PV neurons throughout the vocal control nuclei. We observed differences in EGR1 expression between two classes of PV interneurons in the basal ganglia nucleus Area X. Additionally, we found that singing altered the amount of PV expression in neurons in HVC and Area X and that distinct PV interneuron types in Area X exhibited different patterns of modulation by singing. These data indicate that throughout the vocal control circuitry the singing-related regulation of EGR1 expression in PV neurons may be less influenced by social context than in other neuron types and raise the possibility of cell-type specific differences in plasticity and calcium buffering.

## Introduction

Vocal signals are critical for communication across a range of species, and their production and performance can be modulated by social cues [1]. For example, the content and structure of signals can be influenced by the presence of kin [2,3], territory invaders [4,5], familiar conspecifics [6], and potential mates [7–10]. However, although it is clear that the audience for communication signals can rapidly modulate signal performance, relatively little is known about how social context influences the nervous system to alter signal production.

**Fig 1:**
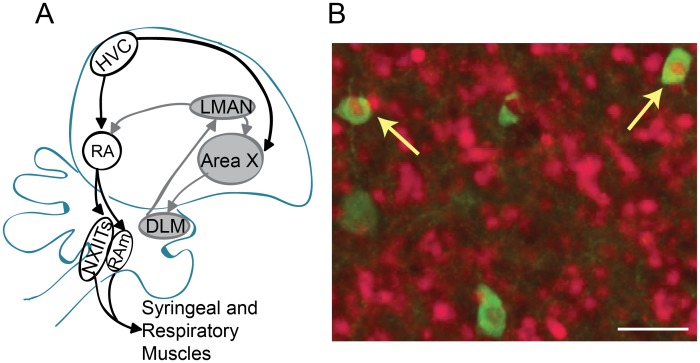
Diagram of the connections in songbird vocal control circuitry. Illustrated are HVC (proper name) and the robust nucleus of the arcopallium (RA) in the vocal motor pathway (white circles) and the basal ganglia nucleus Area X, the dorsolateral anterior thalamic nucleus (DLM), and the cortical nucleus, the lateral magnocellular nucleus of the anterior nidopallium (LMAN) in the anterior forebrain pathway (gray circles). (B) Photomicrograph of PV neurons (green, 488 filter) and EGR1 neurons (red; 596 filter) in Area X during undirected singing. Yellow arrows indicate colocalization. White scale bar = 25 μm.

Songbirds offer a powerful opportunity to study the neural mechanisms underlying the social modulation of communication signals. In zebra finches, males naturally produce songs in distinct social contexts. In particular, they perform a self-initiated song when alone (undirected or UD song) and a courtship song when exposed to a female (female-directed or FD song; [10–13]). Song learning and production are dependent on two specialized circuits dedicated singing (Fig 1A). One pathway, known as the vocal motor pathway (VMP), is analogous to cortical motor circuits in mammals and encodes the motor commands for song [14–17]. The VMP includes forebrain areas such as the nucleus HVC (used as proper name) and the robust nucleus of the arcopallium (RA; [14]). A second pathway, known as the anterior forebrain pathway (AFP), is an avian forebrain-basal ganglia circuit that is homologous to cortical-basal ganglia circuits in mammals and important for song learning and plasticity [18,19]. The AFP consists of the basal ganglia nucleus Area X, the dorsolateral anterior thalamic nucleus (DLM), and the cortical nucleus, the lateral magnocellular nucleus of the anterior nidopallium (LMAN). The basal ganglia nucleus within the AFP, Area X, shows considerable homology with the mammalian basal ganglia, including the presence of neuron types with similar molecular signatures and activity patterns as those described in the mammalian striatum and pallidum [20–25].

Neurons within the VMP and AFP exhibit singing-related changes in activity and immediate early gene expression that have been shown to be modulated by social context [11,26–33]. For example, neurons in LMAN and Area X have higher firing rates and greater expression of EGR1 mRNA and protein during UD singing than FD singing [11,28–39]. One challenge, however, is that nuclei in both the VMP and AFP consist of different types of neurons, and it remains less understood to what degree specific neuronal populations across the pathways contribute to the socially-modulated changes in song. Here, we investigated the degree to which EGR1 expression within a specific subpopulation of neurons differentially responds to changes in social context (Fig 1B). We focused on neurons expressing parvalbumin (PV), a slow calcium buffer found in GABAergic neurons throughout the brain. Local inhibitory networks involving PV neurons are critical parts of sensory and motor networks that regulate the activity and output of neural circuits [34–36]. Thus, understanding the degree to which EGR1 expression in these neuron types are modulated by singing or social context can lend insight into the circuit dynamics in these nuclei.

## Materials and methods

### Animals

Adult male zebra finches (n = 18, >120 days old) were either hatched and raised in a breeding colony at McGill University or were obtained from an outside vendor (Ontario, Canada). Prior to the start of the experiment, birds were socially housed with same-sex conspecifics, kept on a 14L:10D photoperiod, and provided finch seed, grit, and water *ad libitum*. All procedures were in accordance with the animal care protocols approved by the McGill University Animal Care and Use Committee (#2011–5983) as well as with guidelines of the Canadian Council on Animal Care.

### Experimental procedure

Males were divided into three groups: female-directed (FD) singers (n = 6), undirected (UD) singers (n = 6) and non-singing controls (n = 6). At least 48 hours prior to experimentation, each male was housed individually in a cage inside a sound isolation chamber (‘soundbox’; TRA acoustics, Ontario, Canada) containing a microphone and a video camera connected to a video monitor. The output from the microphone was digitally recorded using a song-activated recording system (Sound Analysis Pro; 44.1 kHz; [37]). On the day of the experiment (‘experimental day’), we began monitoring individuals immediately after the lights turned on. We collected FD songs in a manner similar to previous studies (e.g., [11,32,38]). Briefly, after the lights turned on in the morning of the experimental day, males were given a few minutes to eat and drink before starting female presentations. To collect FD song, we introduced a small cage containing a female into the soundbox. Females remained in the soundbox until males performed one bout of FD song or up to one minute, whichever happened first. Females were then briefly removed, for one to five minutes, and presented again. Males were prevented from singing UD song between female presentations by gently tapping on the soundbox whenever males attempted to sing [32,38]. Testing was stopped if a male performed any UD song between female presentations and then restarted on the following day. If a male failed to sing to a particular female on more than one presentation, we subsequently exposed the male to a different female to increase the probability of eliciting FD song. In all cases, we monitored the duration of singing during each female presentation and aimed to collect at least two minutes of FD song in a one-hour period (average: 2.0 minutes, range: 1.8–2.3 minutes; [11,31,32,38]).

Because EGR1 expression co-varies with the amount of song produced [11,39], we “yoked” the amount of UD song to the amount of FD song. To this end, we collected our data as pairs of birds producing FD (n = 6) or UD song (n = 6) and used the amount of FD song produced by the FD bird of the pair as the limit for the amount of UD song for the UD bird of the pair. Once the UD singer of the pair reached the limit set by the FD singer of the pair, we turned off the lights in the soundbox housing the UD singer to prevent further singing. The total amount of song produced by FD and UD singers was not significantly different (F_(1,5)_ = 0.3, p = 0.6268; mixed-effects ANOVA (independent variable: context (FD vs. UD), random effect: pair identity, i.e. which birds were ‘yoked’)).

Non-singing birds (n = 6) were maintained in a soundbox with the lights on for at least one hour after lights came on in the morning. To prevent singing during this time, we kept the soundbox door slightly ajar and gently tapped on the soundbox whenever birds attempted to sing (e.g., [11,31,32,40,41]). All experiments were conducted in the morning, beginning shortly after lights turned on. To limit potential circadian effects on song and neural activity, if a male did not perform sufficient amounts of FD or UD song within three hours of lights on we ended the experiment and repeated it the following day.

### Brain collection and immunocytochemistry

Once males had performed enough song, the lights were turned off to prevent further singing, and males remained undisturbed in the dark [32,38]. Ninety minutes after the start of their first song bout, subjects were deeply anaesthetized with isofluorane vapor and transcardially perfused with heparinized saline (100 IU/100mL) followed by 150 mL of 4% paraformaldehyde (PFA; pH 7.4). Brains were collected and post-fixed for 4 hours in PFA then sunk in 30% sucrose. We cut 40 μm, sagittal sections using a sliding microtome (Leica Biosystems, Wetzlar, Germany), and stored sections in 0.025M phosphate-buffered saline (PBS) with sodium azide at 4°C until processing.

Brain sections were divided into six immunocytochemical batches, each of which contained sections for one FD, one UD, and one non-singing bird. Immunocytochemistry was performed on every third section as previously described [13,32,38,42,43]. Briefly, sections underwent 3 X 10 minute rinses in 0.025M PBS followed by a 1 h incubation in 5% donkey serum and 0.3% Triton-X. Following another set of washes (3 X 10 minute), sections were incubated for 48 h at 4°C in rabbit anti-EGR1 (1:1000 dilution; Santa Cruz Biotechnology, Santa Cruz, CA, USA) and mouse monoclonal PV (clone PARV-19; 1:1000 dilution; Sigma Aldrich, St. Louis MO, USA). Sections were then rinsed 2 X 10 minutes in 0.025M PBS and incubated for 2 h in donkey anti-rabbit secondary antibody conjugated to Alexa Fluor 568 (5μl/ml) and donkey anti-mouse secondary antibody conjugated to Alexa Fluor 488 (5μl/ml). Sections were rinsed in 0.025M PBS and mounted and cover-slipped (ProLong Gold Antifade Reagent, Life Technologies, Carlsbad, CA, USA) on aluminum-chromium subbed slides.

### Imaging

Photomicrographs were taken of each nucleus in each hemisphere using a 20X objective on a Zeiss Axio Imager upright microscope and an AxioCam MRm Zeiss camera (Carl Zeiss, Jena, Germany; image frame resolution: 1388 x 1040 pixels). Separate monochrome images for EGR1 and PV expression were obtained from each section and then color-coded in the ZEN imaging software (Carl Zeiss, Jena, Germany). Sections were imaged according to immunocytochemical batches, each containing sections from one FD, one UD and one non-singing bird. For each region of interest, we determined the exposure times for each bird in a batch using the 20X objective then calculated the average exposure time for each fluorescence wavelength channel (488 and 568 um). The average exposure time across all birds in the batch was used to take photomicrographs for the region of interest.

For counts of EGR1-positive and PV-positive neurons, images were imported into Photoshop (Adobe) and cropped to a specified window size for quantification. We used a 0.20 x 0.20 mm window for HVC, RA, and Area X, and a 0.15 x 0.15 mm window for LMAN. Windows were positioned in the center of each nucleus, ensuring that the entire window fit within the borders of the brain area. For each nucleus, we counted cells on all sections on which we could fit the counting window (3.89 ± 0.06, mean ± SEM). For HVC, we quantified cells in the three most medial sections (medial HVC) and the three most lateral (lateral HVC) sections. We manually counted the number of EGR1-positive and PV-positive neurons as well as the number of PV-positive neurons expressing EGR1 for each image (Fig 1B). We calculated and analyzed the density of EGR1- positive and PV-positive cells.

We also measured the relative amount of PV within PV-positive cells. Levels of PV can be modulated by activity [44,45] and measuring the relative intensity of fluorescence can provide an indication of relative protein expression levels [46–52]. To measure luminance, we took photomicrographs of the entire nucleus on two (HVC, RA, LMAN) or four (Area X) tissue sections. For each section, all PV-positive neurons were outlined using Fiji imaging software [53]. We then measured the integrated fluorescence density (IFD), which is the sum of all pixel intensities within a defined area, for PV. We also measured the IFD of a background area of similar size, located adjacent to each cell and devoid of fluorescent objects. We calculated the mean IFD for the background, multiplied that by the size of the outlined PV cell, then subtracted the background IFD value from the IFD for the PV cell. The background subtracted IFD was then divided by the total area to calculate the corrected total cellular fluorescence density [54,55]. We refer to this background subtracted measure as “luminance”. In total, we measured luminance in 363 cells in medial HVC, 441 cells in lateral HVC, 1363 cells in RA, 644 cells in LMAN and 2194 cells in Area X.

In Area X, two types of PV-positive neurons—fast-spiking interneurons (FSI) and external globus pallidus (GPe) neurons—have been identified and can be differentiated based on size [20,25]. To further investigate EGR1 and PV in these neurons, we divided cells into two classes based on cell size. Neurons between 7–11 μm in diameter were categorized as putative FSI neurons, and neurons 13–14 μm in diameter were categorized as putative GPe neurons [20,25]. We manually counted the number of PV-positive neurons expressing EGR1 and the regulation of PV in both neuron types in Area X. All imaging and quantification were performed by individuals blind to the experimental group.

### Statistical analyses

The distribution of counts of EGR1-positive neurons and PV-positive neurons, and the percentage of PV-positive neurons expressing EGR1 were not normally distributed and various transformations failed to normalize the distribution. Therefore, data for each brain area and for different cell types within Area X were analyzed using non-parametric statistics [56]. For these analyses, data were blocked by immunocytochemical (ICC) batch (mean-centered) in order to account for variation across batches. We performed Wilcoxon rank sum tests with context (NS, FD singing or UD singing) as the independent variable and the density of EGR1-positive neurons, the density of PV-positive neurons, or the percent of PV-positive neurons expressing EGR1 as the dependent variables. The Steel-Dwass method, which represents a non-parametric equivalent of the Tukey’s HSD test, was used for post-hoc contrasts. To analyze variation between the cell types within Area X, we also performed Wilcoxon rank sum tests within each context with cell-type as the independent variable and percent of neurons expressing EGR1 as the dependent variable.

The data for PV luminance for neurons in all brain areas were normally distributed; consequently, we used a mixed-effects ANOVA with context as the independent variable and the average PV luminance for each bird as the dependent variable. For the analysis of PV luminance within FSI and GPe neurons in Area X, we used a two-way, full factorial mixed-effects ANOVA with context, cell type and the interaction as independent variables, and the average luminance of PV for each cell type for each bird as the dependent variable. For all mixed models, we included ICC batch as a random variable to control for variation between batches. For the analysis of FSI and GPe neurons, we also included individual ID nested in ICC batch as a random variable to control for the fact that both FSI and GPe neurons were measured from each individual. For the analysis of luminance we used Tukey’s HSD for post-hoc comparisons.

All statistics were performed using JMP 11.2.0 (Cary, NC, USA), and α = 0.05 for all analyses.

## Results

### Effect of singing and social context on EGR1 expression

Both singing and the social context in which song is produced have previously been found to affect EGR1 expression throughout song control nuclei in various songbirds, including zebra finches [11,38,40,41]. Here, we first confirmed that singing and social context modulated EGR1 protein expression in HVC, RA, Area X, and LMAN, and then analyzed the expression of EGR1 and PV within PV-positive neurons in each brain area.

**Fig 2:**
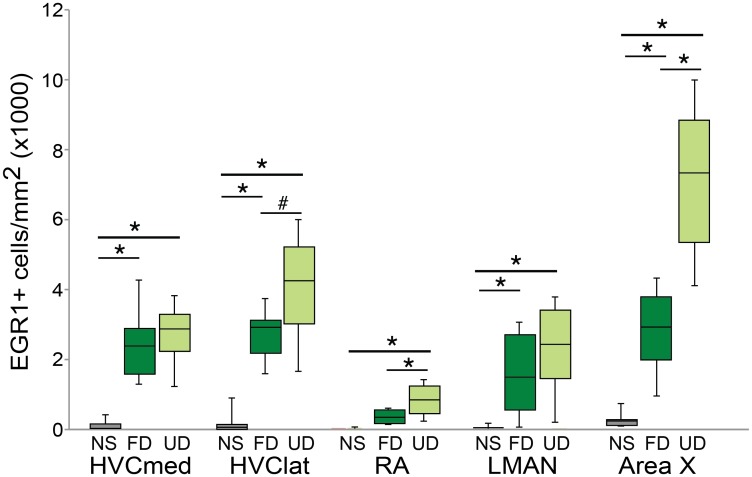
Modulation of EGR1 protein by singing and social context varies across the song system. EGR1 expression among non-singing birds (NS; gray bars), birds producing female-directed song (FD; dark green bars) and birds producing undirected song (UD; light green bars). Box-and-whisker plots for each experimental group. Each box spans the interquartile range, horizontal black lines indicate the median and whiskers show the minima and maxima. Lines above bars indicate significance of post-hoc contrasts for brain areas in which experimental groups significantly differed. * indicates a significant difference at p<0.05; # indicates a trend toward a difference at p<0.10.

Previous studies have found differences in EGR1 expression between the medial and lateral portions of HVC (e.g., [11]). As such, we independently analyzed context-dependent changes in EGR1 expression in the medial and lateral portions of HVC (HVC-med and HVC-lat, respectively). EGR1 expression was significantly different across experimental groups in both HVC-med (χ^2^_2_ = 12.3, p = 0.0021; Fig 2) and HVC-lat (χ^2^_2_ = 14.0, p = 0.0009; Fig 2). In both portions of HVC, EGR1 expression was significantly higher for birds producing either UD song or FD song than for non-singing birds (p<0.05 for all contrasts). In HVC-lat but not in HVC-med, there was a trend for EGR1 expression to be higher in birds producing UD song than in birds producing FD song (p = 0.0528).

Social context affected EGR1 expression in the nucleus RA (χ^2^_2_ = 12.8, p = 0.0017; Fig 2). EGR1 expression in RA was significantly higher in birds producing UD song than in either non-singing birds or birds producing FD song (p<0.05 for each contrast). There was a trend for EGR1 expression to be higher in birds producing FD song than in non-singing birds (p = 0.0779).

Social context also affected the expression of EGR1 in Area X (χ^2^_2_ = 15.2, p = 0.0005; Fig 2). EGR1 expression was significantly higher in birds producing UD song than in either non-singing birds or birds producing FD song (p<0.0150 for each contrast). Additionally, EGR1 expression was significantly higher in birds producing FD song than in non-singing birds (p<0.0150).

In LMAN, there was a significant effect of singing but not of social context on EGR1 expression (χ^2^_2_ = 11.1, p = 0.0039; Fig 2). Birds producing UD song or FD song had more EGR1 expressing neurons than non-singing birds (p<0.05 for both contrasts), however, there was no difference in EGR1 expression between birds producing UD song or FD song (p = 0.4526).

### Effects of singing on EGR1 expression in PV-positive neurons

**Fig 3:**
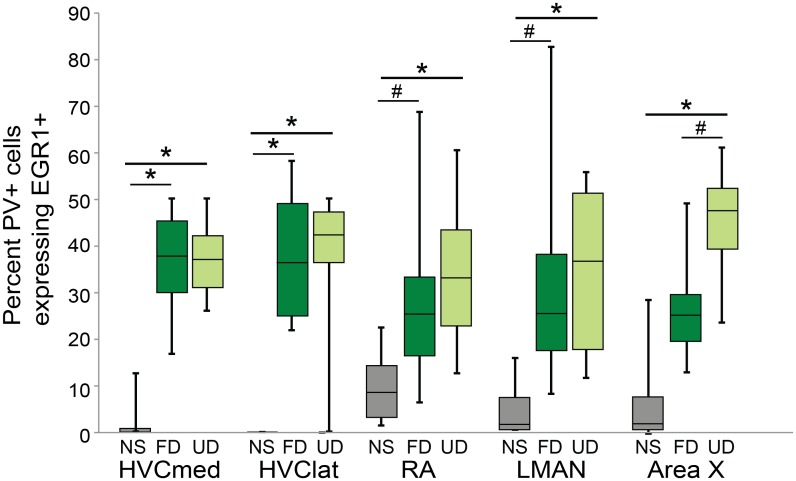
Percent of PV neurons expressing EGR1 across nuclei is modulated by singing. Percent of PV neurons expressing EGR1 in non-singing birds (NS; gray bars), birds producing female-directed song (FD; dark green bars) and birds producing undirected song (UD; light green bars). Box-and-whisker plots for each experimental group. Each box spans the interquartile range, horizontal black lines indicate the median and whiskers show the minima and maxima. Lines above bars indicate significance of post-hoc contrasts for brain areas in which experimental groups significantly differed. * indicates a significant difference at p<0.05; # indicates a trend toward a difference at p<0.10.

Parvalbumin neurons are significant components of local inhibitory networks and important for regulating the activity and output of neural circuits [57–60]. Here, we investigated whether EGR1 expression in PV-positive subpopulations was modulated by singing and social context. Specifically, we analyzed the degree to which singing and social context affected the percent of PV-positive neurons that express EGR1 within HVC, RA, LMAN and Area X (Fig 3).

In HVC, the proportion of PV-positive neurons expressing EGR1 was significantly different across experimental groups (HVC-med: χ^2^_2_ = 9.4, p = 0.0092; HVC-lat: χ^2^_2_ = 11.1, p = 0.0038; Fig 3). In both the medial and lateral portions of HVC there were significant effects of singing, with more PV-positive neurons expressing EGR1 in birds producing either UD or FD song than in non-singing birds (p<0.05 for all contrasts). In contrast, in neither portion of HVC was there a significant difference in EGR1 expression in PV-positive neurons between birds producing UD or FD song (p>0.95).

The proportion of PV-positive neurons in RA expressing EGR1 was significantly different across experimental groups (χ^2^_2_ = 8.1, p = 0.0176; Fig 3). Significantly more PV-positive neurons expressed EGR1 in birds producing UD song than in non-singing birds (p = 0.0349). The proportion of PV neurons expressing EGR1 in FD singers was intermediate, with slightly but not significantly greater EGR1 expression compared to non-singing birds (p = 0.0779) and similar expression to UD singers (p = 0.9155).

The proportion of PV-positive neurons in LMAN that expressed EGR1 was also significantly different across experimental groups (χ^2^_2_ = 8.3, p = 0.0156; Fig 3). Significantly more PV-positive neurons expressed EGR1 in birds producing UD song than in non-singing birds (p = 0.0349), and there was a trend for more PV-positive neurons to express EGR1 in birds producing FD song than in non-singing birds (p = 0.0779). The percent of PV-positive neurons expressing EGR1 was not different between UD and FD singers (p = 0.7513).

EGR1 expression in PV-positive neurons in Area X was also different across the experimental groups (χ^2^_2_ = 11.3, p = 0.0036; Fig 3). In particular, significantly more PV-positive neurons expressed EGR1 in birds producing UD song than in non-singing birds (p = 0.0224). EGR1 expression in PV neurons of birds singing FD song was intermediate, with a trend towards higher EGR1 expression compared to non-singing birds (p = 0.1118) and lower EGR1 expression compared to birds singing UD song (p = 0.0528).

**Fig 4:**
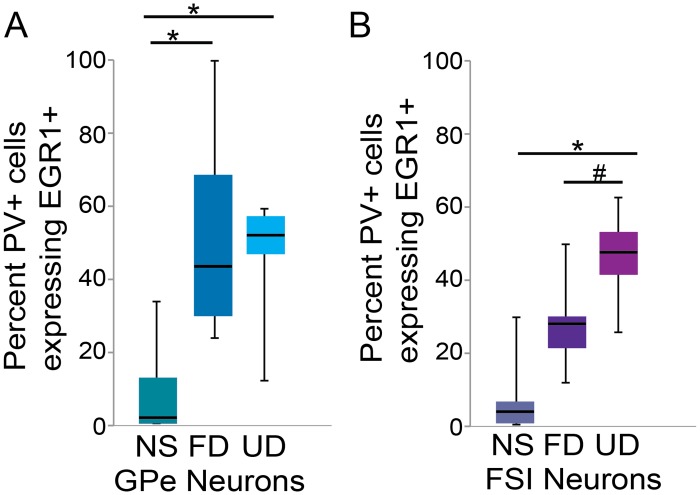
Singing and social context differentially affect the percent of neurons expressing EGR1 in two types of Area X interneurons. The percent of external pallidal neurons (GPe; A) expressing EGR1 is modulated by singing. In fast-spiking interneurons (FSIs; B), the proportion of cells expressing EGR1 is highest in birds singing UD song, lowest in non-singing birds, and intermediate in birds singing FD song. Moreover, in birds singing FD song, a greater proportion of GPe neurons than FSI neurons express EGR1. Box-and-whisker plots for each experimental group. Each box spans the interquartile range, horizontal black lines indicate the median and whiskers show the minima and maxima. * indicates a significant difference at p<0.05, # indicates a trend toward a difference at p<0.10.

Previous studies have identified two different subtypes of PV neurons in Area X that appear to correspond to fast-spiking interneurons (FSIs) and putative external globus pallidus neurons (GPe)[20,25], and we investigated the degree to which these distinct PV cell types could be differentially modulated by singing and social context. The percent of FSI and GPe neurons expressing EGR1 (see Methods) was significantly different across experimental groups (GPe neurons: χ^2^_2_ = 9.7, p = 0.0078; FSIs: χ^2^_2_ = 10.9, p = 0.0043). In GPe neurons, EGR1 was modulated by singing, but not by social context (Fig 4B). In particular, a greater percent of GPe neurons expressed EGR1 in both UD singers (p = 0.0349) and FD singers (p = 0.0224) relative to non-singing birds, and the percent of GPe neurons expressing EGR1 was not significantly different between UD and FD singers (p>0.50). Similarly, EGR1 expression in FSI neurons, was significantly higher in UD singers relative to non-singing birds (Fig 4A; p = 0.0224). Expression in FD singers was intermediate, with a trend towards lower expression relative to UD singers (p = 0.0779) and toward higher expression relative to non-singing birds (p = 0.1118). We also compared the percent of PV neurons expressing EGR1 between the two cell types within each behavioral context. We found that the percent of GPe neurons expressing EGR1 was significantly higher than the percent of FSI neurons expressing EGR1 in birds producing FD song (χ^2^_2_ = 5.0, p = 0.0250) but not in UD singers or non-singing birds. Thus, while EGR1 expression in both neuron types is increased during UD singing, the degree of EGR1 expression during FD singing depended on the cell type.

### Regulation of PV expression across the song system

**Fig 5:**
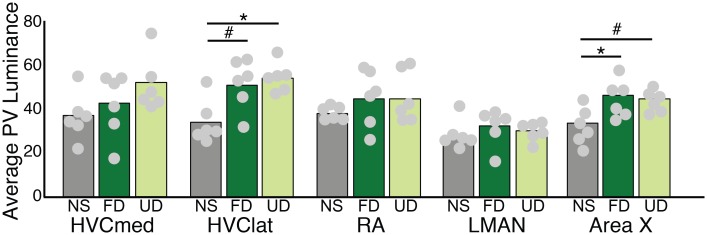
Parvalbumin (PV) luminance is modulated by singing in HVC and Area X. PV luminance in HVC-lat and Area X differed between non-singing birds (NS; gray bars) and singing birds (female-directed singers, FD; dark green bars; undirected singers, UD; light green bars). Bars represent means, while circles correspond to individual data points. * indicates a significant difference at p<0.05, # indicates a trend toward a difference at p<0.10.

The amount of PV can be affected by activity [44,45], so we also investigated whether singing and social context affected relative PV levels. There were significant differences in PV luminance across experimental groups in HVC-lat (F_(2,10)_ = 5.4, p = 0.0264) and Area X (F_(2,10)_ = 9.7, p = 0.0045), but not in HVC-med, RA or LMAN (Fig 5). In general, in both HVC-lat and Area X, there were effects of singing but not of social context. In HVC-lat, PV luminance was significantly higher in UD singers than in non-singing birds (p = 0.0349), and there was a trend toward higher PV luminance in FD singers than in non-singing birds (p = 0.0528). However, there was no significant difference in PV luminance between UD and FD singers (p = 0.8805). In Area X, PV luminance in both UD (p = 0.0046) and FD (p = 0.0224) singers was significantly higher than in non-singing birds but not different between UD or FD birds (p = 0.6027).

**Fig 6:**
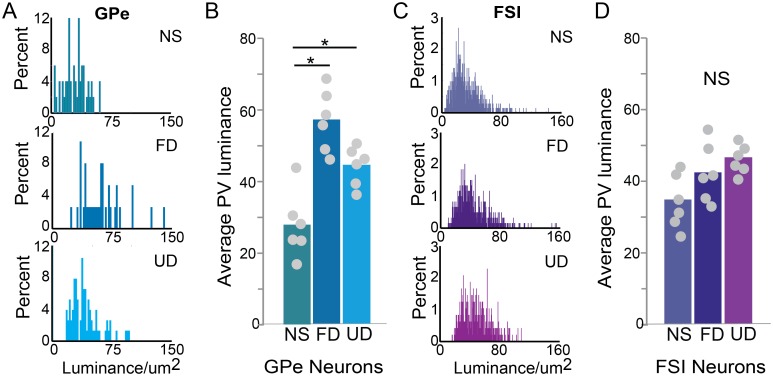
Regulation of PV luminance by singing differs between two interneuron classes. Histograms of the luminance values for all GPe (A) and FSI (C) neurons measured in non-singing birds (top panel), birds singing female-directed song (FD; middle panel), and birds singing undirected song (UD; bottom panel). (B) Average PV luminance in GPe neurons differed between singing (both FD and UD) and non-singing birds (D) Neither singing nor social context affected PV luminance in FSI neurons. For (B) and (D), bars represent means while circles correspond to individual data points. * indicates a significant difference at p<0.05.

Interestingly, while EGR1 regulation was generally similar between FSI and GPe neurons (Fig 4), the regulation of PV varied across these neuron types (F_(2,15)_ = 9.7, p = 0.0020). We quantified the average luminance of PV (see Methods) for each cell type and found that PV luminance was significantly different across experimental groups for GPe neurons (Fig 6A and 6B) but not FSIs (Fig 6C and 6D). For GPe neurons, PV luminance was significantly higher in birds producing FD songs (p = 0.0003) and in birds producing UD songs (p = 0.0453) than in non-singing birds and was not significantly different between birds producing FD or UD song (p = 0.1838).

Variation between cell types was also evident within experimental conditions. In particular, PV luminance was significantly higher in GPe neurons than in FSI neurons in FD singers (p = 0.0110) but not in UD singers or non-singing birds.

## Discussion

In both the vocal motor pathway (VMP) and anterior forebrain pathway (AFP) of songbirds, singing and social context have been shown to modulate neural activity and immediate early gene expression [11,28–30,33,61]. Here, we tested whether parvalbumin (PV) expressing subpopulations within the VMP and AFP show similar social modulations of EGR1 expression relative to EGR1 expression as a whole. We found that EGR1 expression in PV neurons throughout the song system was generally modulated by singing but not by social context. In addition, we also observed that PV expression was modulated by singing in HVC and Area X. Finally, particular PV neuron types in Area X differed in the degree to which EGR1 and PV were modulated by singing. These data indicate that EGR1 expression may be differentially modulated within particular neuron subpopulations and suggest distinct contributions of different cell types to the social modulation of neural activity and behavior.

Our results for overall EGR1 expression generally parallel those that have been previously reported, with greater increases in EGR1 expression during UD than FD singing. As in a previous study [11], EGR1 expression in HVC was increased during both UD and FD singing, and social context did not significantly affect EGR1 expression, though there was a trend for higher EGR1 in UD singers in lateral HVC. However, our results differ from previous studies with regard to EGR1 expression during FD singing. In particular, whereas previous studies did not find an increase in EGR1 mRNA expression in Area X and LMAN during FD singing [11,41], we observed that EGR1 protein expression in Area X and LMAN increased significantly during FD singing compared to non-singing. Similarly, in RA there was a trend toward higher EGR1 protein expression during FD singing compared to non-singing. The increases in EGR1 protein expression with FD singing are similar to those described in Bengalese finches [32]. One possible explanation may lie in differences between mRNA and protein expression, as both our study and [32] investigated protein expression while [11] and [41] studied mRNA expression. Indeed, previous studies have found a complex regulation of EGR1 transcription and translation with singing in song control nuclei [62,63].

EGR1 expression is not only used as a cellular marker of activity but also a marker of plasticity [64–66]. Immediate early genes like EGR1 are transcription factors that influence the expression of a variety of genes that can affect neuronal function [64,67]. Undirected song has been argued to represent a state of vocal practice and EGR1 expression, overall, is higher in the AFP and RA during UD than FD song [11,68–70]. In contrast, PV-positive neurons have been shown to exhibit more stable synaptic transmission than other types of interneurons, and plasticity of PV neurons in adults is thought to be limited, for example, by the presence of perineuronal nets (PNNs; [71,72]). Moreover, in songbirds, increases in PNNs around PV-positive neurons coincide with the close of the sensitive period for song learning [73], which raises the possibility that songbird PV neurons may also be generally less plastic than other neuron types. Consistent with the idea that PV neurons may be more stable than other neurons, we found that there was no significant social modulation of EGR1 expression in PV neurons in HVC, LMAN, RA and Area X. These data imply that the plasticity of PV neurons might not be modulated by social context, but additional measures of cellular and synaptic plasticity are important to test this hypothesis.

In both medial and lateral HVC a greater percentage of PV-positive neurons expressed EGR1 during singing than non-singing, however there was no social modulation of EGR1 expression. Previous work, as well as our own data presented here, highlight a trend towards higher EGR1 expression during UD song than FD song in the lateral portion of HVC (Fig 2; [11]). In contrast to the general trends for EGR-1 expression in HVC, we found that PV neurons did not exhibit a similar trend towards a social modulation of EGR1 expression. Rather, in lateral (and medial) HVC, the percent of PV neurons expressing EGR1 was almost identical between FD and UD singers. HVC contains a number of additional neuron types, including neurons that express calcium binding proteins other than PV [74], and it will be interesting to reveal the specific neuron subtype(s) in lateral HVC that contribute to the trend toward higher EGR1 expression during UD singing.

We also discovered behaviorally regulated differences in the expression of PV in lateral HVC and GPe neurons in Area X. In both areas, PV expression was modulated by singing (Figs 4 and 5). Calcium buffering proteins, including PV, modulate the size and duration of calcium transients, thereby altering synaptic plasticity and homeostasis as well as the linearity of information transfer at synapses [34,35,75–77]. Given the intrinsic and singing-related differences in PV, additional studies of calcium and calcium buffering at shorter time-scales may uncover critical information on the roles of these PV neurons in modulating circuit activity. In addition, given known differences in the singing-related firing patterns of neurons within Area X, LMAN, RA and HVC it is unclear what leads to differences in PV expression in some neurons but not others. Further investigation will be necessary to uncover what contributes to such cell-type specific differences in the regulation of calcium and calcium buffering proteins.

In mammals, there are at least three types of GPe neurons that have been distinguished based on their projections, firing patterns, and gene expression [78–80]. One, known as the prototypic neuron type, expresses PV, GABA, and the homeobox gene Nkx2.1 and projects primarily to the subthalamic nucleus [78–80]. We hypothesize, based on the similarity in PV expression, that our songbird GPe neurons correspond to this prototypical mammalian type [20,25]. However, little is known about whether the other two types, an Lhx6-expressing neuron that also projects to the subthalamic nucleus and an “arkypallidal” type that expresses preproenkephalin and projects to the striatum, are present in songbird Area X. Determining the targets and connections of large, PV-positive neurons in Area X will be necessary to further classify these neurons as similar to prototypical mammalian GPe neurons and will lend further insight into the circuit level similarity between songbird Area X and mammalian basal ganglia.

In both FSI and GPe neurons, the highest levels of EGR1 expression occurred in birds that performed undirected song. While EGR1 expression was also increased during FD singing, the degree of increase was greater in GPe than FSI neurons. However, in neither cell type was there a significant effect of social context. The lack of an effect of social context on EGR1 expression contrasts with the substantial social modulation of firing rate, precision and bursting of both cell types recorded during singing [61]. While EGR1 is often used as a proxy of neural activity, these data highlight that the relationship between EGR1 regulation and firing is likely more complex. In particular, these data support the possibility that there may be discrete roles or functions of molecular activity markers and electrophysiological signals in these neuron types.

While FSI and GPe neurons had similar EGR1 expression patterns, we found intrinsic and behaviorally regulated differences in the level of PV expression between the neuron types. In particular, unlike FSI neurons, GPe neurons exhibited low PV expression during non-singing as well as singing-related increases in PV expression. The PV antibody used here binds only to calcium bound PV, thus, the difference in PV expression may reflect differences in the level of calcium or degree of calcium binding between the cell types. Dynamic regulation of calcium critically influences activity and communication within neural circuits through effects on synaptic plasticity and homeostasis [75,77]. By modulating the time course and amplitude of calcium transients, calcium buffers contribute to this regulation and may thereby alter or maintain synaptic strength and plasticity [76]. Whether the differences that we found in PV expression relate to cell type differences in excitability, synaptic strength and plasticity or to changes in excitatory or neuromodulatory inputs is unknown. Determining what regulates the changes in PV expression of these two neuron types could lend critical insight into their roles in modulating circuit activity.

## Supporting information

S1 Data Mean counts and luminance values for PV and EGR1 neurons throughout the song system. (TXT)

S2 Data Mean counts and luminance values for PV and EGR1 in Area X FSI and GPe neurons. (TXT)
